# Supplementary material for: Social isolation recruits amygdala–medial prefrontal cortex projections to escalate alcohol drinking in male mice
Source: Nat Neurosci. 2026 Aug 25;29(10):2513–22. doi: 10.1038/s41593-026-02413-x (PMC13633117; doi:10.1038/s41593-026-02413-x)
Supplement: Supplementary file 1 [file 41593_2026_2413_MOESM1_ESM.pdf]

nature portfolio

Corresponding author(s): Reesha R. Patel and Kay M. Tye

Last updated by author(s): Jun 12, 2026

Reporting Summary

Nature Portfolio wishes to improve the reproducibility of the work that we publish. This form provides structure for consistency and transparency in reporting. For further information on Nature Portfolio policies, see our Editorial Policies and the Editorial Policy Checklist.

Statistics

For all statistical analyses, confirm that the following items are present in the figure legend, table legend, main text, or Methods section.

n/a	Confirmed	
☐	☒	The exact sample size (*n*) for each experimental group/condition, given as a discrete number and unit of measurement
☐	☒	A statement on whether measurements were taken from distinct samples or whether the same sample was measured repeatedly
☐	☒	The statistical test(s) used AND whether they are one- or two-sided<br>*Only common tests should be described solely by name; describe more complex techniques in the Methods section.*
☒	☐	A description of all covariates tested
☐	☒	A description of any assumptions or corrections, such as tests of normality and adjustment for multiple comparisons
☐	☒	A full description of the statistical parameters including central tendency (e.g. means) or other basic estimates (e.g. regression coefficient) AND variation (e.g. standard deviation) or associated estimates of uncertainty (e.g. confidence intervals)
☐	☒	For null hypothesis testing, the test statistic (e.g. *F*, *t*, *r*) with confidence intervals, effect sizes, degrees of freedom and *P* value noted<br>*Give P values as exact values whenever suitable.*
☒	☐	For Bayesian analysis, information on the choice of priors and Markov chain Monte Carlo settings
☒	☐	For hierarchical and complex designs, identification of the appropriate level for tests and full reporting of outcomes
☐	☒	Estimates of effect sizes (e.g. Cohen's *d*, Pearson's *r*), indicating how they were calculated

Our web collection on statistics for biologists contains articles on many of the points above.

Software and code

Policy information about availability of computer code

Data collection	Data were collected with commercially available software including: Nolduls Ethovision XT 14, Med-PC IV 4.2, Inscopix Data Acquisition Software, pClamp10, BZ-X Wide Image Viewer 1.3.1.1
Data analysis	Data were analyzed with commercially available, open-sourced, and custom code and sofware including: Inscopix Data Processing Software, Matlab 2025b, Figi, SLEAP, Clampfit v.11 BZ-X Wide Image Analyzer 1.3.1.1, Graphpad Prism 10.Descriptions of these analyses are found in the methods. In cases that there are published descriptions of the methods, full references are included.

For manuscripts utilizing custom algorithms or software that are central to the research but not yet described in published literature, software must be made available to editors and reviewers. We strongly encourage code deposition in a community repository (e.g. GitHub). See the Nature Portfolio guidelines for submitting code & software for further information.

Data

Policy information about availability of data

All manuscripts must include a data availability statement. This statement should provide the following information, where applicable:

- Accession codes, unique identifiers, or web links for publicly available datasets
- A description of any restrictions on data availability
- For clinical datasets or third party data, please ensure that the statement adheres to our policy

All data supporting the findings of this study are available in the main text, supplementary materials, and the source data files provided with this paper. Unprocessed data have been deposited in the DANDI Archive under accession code 001454 (https://dandiarchive.org/dandiset/001454).

Research involving human participants, their data, or biological material

Policy information about studies with human participants or human data. See also policy information about sex, gender (identity/presentation), and sexual orientation and race, ethnicity and racism.

Reporting on sex and gender	n/a
Reporting on race, ethnicity, or other socially relevant groupings	n/a
Population characteristics	n/a
Recruitment	n/a
Ethics oversight	n/a

Note that full information on the approval of the study protocol must also be provided in the manuscript.

Field-specific reporting

Please select the one below that is the best fit for your research. If you are not sure, read the appropriate sections before making your selection.

☒ Life sciences ☐ Behavioural & social sciences ☐ Ecological, evolutionary & environmental sciences

For a reference copy of the document with all sections, see nature.com/documents/nr-reporting-summary-flat.pdf

Life sciences study design

All studies must disclose on these points even when the disclosure is negative.

Sample size	No statistical methods were used to pre-determine sample sizes. Sample sizes were based on those commonly used in the field for similar rodent behavioral and circuit-level experiments, including fiber photometry, optogenetic manipulation, and two-bottle choice paradigms. These sample sizes are consistent with effect sizes typically observed in such experiments. Sample sizes for each experiment are reported in the figure legends.
Data exclusions	Animals were excluded based on histological verification. Mice with less than 50 licks at the alcohol bottle were excluded from analysis for calcium recordings to ensure enough trials.
Replication	The main behavioral finding for social isolation induced increased alcohol drinking in males were replicated across three cohorts and females across 2 cohorts. A total of 17 cohorts of mice were used in the experiments.
Randomization	Mice were randomly assigned into experimental and control groups. All mice were handled and underwent the exact same procedures. Stimulation days for resident-intruder were counter balanced across animals. Position of alcohol and water bottles were counter balanced across mice.
Blinding	During behavioral testing investigators were not always blind to the condition (experimental vs. control) given familiarity with the subjects. However, for histology, ex vivo electrophysiology, optogenetic experiments, experimenters were blinded to the group assignment until after data analysis and data need to be segregated into groups.

Reporting for specific materials, systems and methods

We require information from authors about some types of materials, experimental systems and methods used in many studies. Here, indicate whether each material, system or method listed is relevant to your study. If you are not sure if a list item applies to your research, read the appropriate section before selecting a response.

Materials & experimental systems

n/a	Involved in the study
☒	☐ Antibodies
☒	☐ Eukaryotic cell lines
☒	☐ Palaeontology and archaeology
☐	☒ Animals and other organisms
☒	☐ Clinical data
☒	☐ Dual use research of concern
☒	☐ Plants

Methods

n/a	Involved in the study
☒	☐ ChIP-seq
☒	☐ Flow cytometry
☒	☐ MRI-based neuroimaging

Animals and other research organisms

Policy information about studies involving animals; ARRIVE guidelines recommended for reporting animal research, and Sex and Gender in Research

Laboratory animals	Adult, male and female C57/BL6J mice (8-10 weeks old) from Jackson Laboratory were used for all experiments. Mice were housed under a 12-hour reverse light/dark cycle at ambient temperature (22C) and 40–60% relative humidity.
Wild animals	n/a
Reporting on sex	Both male and female mice were included and reported when used.
Field-collected samples	n/a
Ethics oversight	All experimental procedures were carried out in accordance with NIH guidelines and approval of the Salk and Northwestern Institutional Animal Care and Use Committee.

Note that full information on the approval of the study protocol must also be provided in the manuscript.

Plants

Seed stocks	n/a
Novel plant genotypes	n/a
Authentication	n/a
